# Predominant polarity in bipolar disorder and validation of the polarity index in a German sample

**DOI:** 10.1186/s12888-014-0322-8

**Published:** 2014-11-22

**Authors:** Julia Volkert, Kathrin C Zierhut, Miriam A Schiele, Martina Wenzel, Juliane Kopf, Sarah Kittel-Schneider, Andreas Reif

**Affiliations:** Department of Psychiatry, Psychosomatics and Psychotherapy, University of Wuerzburg, Fuechsleinstrasse 15, Wuerzburg, D-97080 Germany; Department of Psychiatry, Psychosomatic Medicine and Psychotherapy Goethe-University, Heinrich-Hoffmann-Straße 10, Frankfurt am Main, D-60528 Germany

**Keywords:** Bipolar disorder, Predominant polarity, Polarity index, Maintenance treatment, Depression, Mania, EBM

## Abstract

**Background:**

A large number of patients with bipolar disorder (BD) can be characterized by predominant polarity (PP), which has important implications for relapse prevention. Recently, Popovic et al. (EUR NEUROPSYCHOPHARM 22(5): 339–346, 2012) proposed the Polarity Index (PI) as a helpful tool in the maintenance treatment of BD. As a numeric expression, it reflects the efficacy of drugs used in treatment of BD. In the present retrospective study, we aimed to validate this Index in a large and well characterized German bipolar sample.

**Methods:**

We investigated 336 bipolar patients (BP) according to their PP and calculated the PI for each patient in order to prove if maintenance treatment differs according to their PP. Furthermore, we analysed whether PP is associated with demographic and clinical characteristics of BP.

**Results:**

In our sample, 63.9% of patients fulfilled criteria of PP: 169 patients were classified as depressive predominant polarity (DPP), 46 patients as manic predominant polarity (MPP). The two groups differed significantly in their drug regime: Patients with DPP were more often medicated with lamotrigine and antidepressants, patients with MPP were more often treated with lithium, valproate, carbamazepine and first generation antipsychotics. However, patients with DPP and MPP did not differ significantly with respect to the PI, although they received evidence-based and guideline-driven treatment.

**Conclusion:**

The reason for this negative finding might well be that for several drugs, which were used frequently, no PI value is available. Nevertheless we suggest PP as an important concept in the planning of BD maintenance treatment.

## Background

In spite of advances in evidence-based drug treatment, bipolar disorder (BD) remains an often recurrent illness causing severe impairments in patients’ daily life. Thus, relapse prevention is the main objective in treatment strategies of BD. Regarding the drug regimen in BD, a patient’s actual symptomatology plays a crucial role. However, for maintenance treatment the individual illness progress and family history (e.g. information about drug responders) must be considered. Residual symptoms, psychiatric comorbidity, and number of prior episodes have been identified as risk factors of recurrence [1]. Additionally, the polarity of the first mood episode seems to be a good predictor of the predominant polarity of subsequent episodes over time [2], i.e. an overall higher number of depressive episodes is observed in patients with first episode depression and vice versa, and thus should be taken into account in treatment planning.

The categorization of bipolar disorder patients (BP) according to predominant polarity might help to optimize relapse prevention. Patients with a depressive predominant polarity (DPP) have a higher risk of further depressive episodes in the future and therefore need a treatment tailored to preventing depressive relapses. In contrast, patients with manic or hypomanic predominant polarity (MPP) require a drug regimen specifically efficient in the prevention of mania. For example, antidepressants should be avoided in patients with MPP due to the risk of triggering manic symptoms [3]. However, not every BP has a clear tendency for one pole of the disorder. Recent research reported that a clear predominant polarity can be observed in roughly half of BP, and that DPP is more prevalent than MPP [4,5].

Several disease specific variables have been associated with the concept of predominant polarity [5,6]. For instance, it has been demonstrated that depressive polarity coincides with a worse prognosis and decreased treatment response compared to manic polarity [7,8]. Furthermore, the later patients with DPP, often initially misdiagnosed as patients suffering from major depression (“hidden bipolars”), receive a correct diagnosis (and an adequate drug treatment, accordingly), the more severe the course of illness [5,9], i.e. patients with DPP are at a higher risk for misdiagnosis and subsequent worse prognosis. Thus, subtyping of BD by using the concept of predominant polarity could improve the planning of clinical care, long-term prevention, and the development of new therapeutic strategies. Furthermore, more homogeneous subgroups might provide the potential to inform biological studies and the search for biomarkers. Therefore, mechanisms involved in the pathophysiology of BD could be studied in a more target-oriented fashion. However, up to now there are no genetic, familial or biomarker studies on predominant polarity in BD, and the biological underpinnings remain unknown. Treatment guidelines refer to the concept of predominant polarity [10], and some authors even suggest that diagnostic criteria of BD should include predominant polarity as a relevant course descriptor for the illness [11,12]. However, the recently published DSM-5 [13] did not add predominant polarity as a course specifier.

Recently, the idea of predominant polarity in BD was introduced as a helpful tool for maintenance treatment. Popovic *et al.* [14] proposed the Polarity Index (PI) as a descriptor for categorizing drug profiles in the relapse prevention of BD. The Polarity Index is a numeric expression of the efficacy profile of a given drug, derived from the ratio of Numbers Needed to Treat (NNT) for the prevention of depression and NNT for the prevention of mania. The NNT expresses the reciprocal of the absolute risk reduction and is calculated based on the results of several randomized placebo-controlled trials [14]. According to the authors, a PI of 1 indicates equal efficacy of a drug in the prevention of manic and depressive episodes alike. Drugs with a PI >1 have a stronger anti-manic prophylactic effect, while drugs with an index <1 are more efficient in preventing depressive episodes. In a second study, Popovic *et al.* provided evidence for the validity of the index in a large sample of BP by calculating a PI value for each patient [15]. They demonstrated that the mean PI values in their bipolar sample were significantly higher in patients with MPP compared to patients with DPP, indicating a stronger antimanic treatment regimen in MPP. The treatment of patients with DPP was characterized by lower mean PI, indicating a better prevention of depressive episodes. Therefore, the authors suggested the PI as a useful numerical expression of evidence-based maintenance treatment in BD and recommended it as a simple concept for physicians, one that is easily remembered and translatable into clinical practice.

Up to now, most studies on predominant polarity were performed by the Barcelona Bipolar Disorder Group [14-17]. Therefore, the aim of the present study was to validate the PI in a clinical setting with a similar study design in a German sample. To do so, we aimed to replicate the results by Popovic *et al.* by examining a well-characterized bipolar sample in order to determine the role of PI in clinical decision-making regarding maintenance treatment in BD [15]. Furthermore, we were interested in associations between demographic or clinical characteristics and predominant polarity in BP.

## Methods

### Sample

All patients were recruited in the context of the Bipolar Disorder Program at the Department of Psychiatry, Psychosomatics and Psychotherapy, University Hospital Wuerzburg. The program provides multidisciplinary treatment (inpatient and outpatient) for BP from Southern Germany. Since the program started in 2009, a database has been built and allows a prospective follow-up of clinical and sociodemographic variables. Up to now, 464 BP have been phenotyped. Inclusion criterion for the database was a diagnosis of bipolar subtype I or II according to DSM-IV TR [18] criteria. Diagnosis was confirmed by application of the Structured Clinical Interview (SCID-I) [19] for DSM-IV and a consensus diagnosis of two psychiatrists. Several clinical variables were obtained from interviews with patients and their relatives, as well as clinical records, including diagnosis BD type, number of previous episodes (depressive, manic, and mixed), age of illness onset, polarity of first episode, lifetime history of psychotic symptoms, former attempted suicide, rapid cycling, and psychiatric comorbidities [attention deficit hyperactivity disorder (ADHD), substance abuse, panic disorder, migraine]. Furthermore, family history of psychiatric disoders and treatment variables [medication, electroconvulsive therapy (ECT)] were assessed. In addition, patients’ life time psychopathological symptoms were assessed via the structural assessment tool Operational Criteria Checklist for Psychotic and Affective Illness (OPCRIT) [20]. All patients provided written informed consent for the collection of their data for research, participation in studies, and subsequent publication. All procedures followed the Declaration of Helsinki in its latest version and were approved by the Ethical Committee of the medical faculty of the University of Wuerzburg.

For the present study, the data of 336 BP (aged 18-74 years, 202 women and 134 men) with a diagnosis of BD-I or BD-II were included in the analysis. The remaining 128 patients in the database could not be analyzed due to unreliable information about number of previous episodes. Predominant polarity was defined in accordance with previous studies [4,14]: MPP was characterized by at least two-thirds of past episodes complying with DSM-IV criteria for manic/hypomanic episodes, and DPP was defined by at least two-thirds of a patient’s past episodes fulfilling DSM-IV criteria for Major Depressive Episode. Undetermined predominant polarity (UPP) describes cases that did not fall into either category [8]. Mixed episodes were recorded as well but were not considered and assigned as a part of depressive or manic polarity. In our sample only six patients had twice as many mixed episodes than depressed and (hypo-) manic episodes. Therefore we did not include a type of predominant mixed polarity in our analyses.

### Polarity index

In order to calculate the PI, the current treatment of each patient was used, irrespective of prescribed dosage. According to Popovic *et al.* [14], the PI values of each drug for maintenance treatment were as follows: 12.09 for risperidone, 4.38 for aripiprazole, 3.91 for ziprasidone, 2.98 for olanzapine, 1.39 for lithium, 1.14 for quetiapine, and 0.40 for lamotrigine. In the case of polypharmacy, a value for each patient’s treatment was calculated as the mean value of all prescribed drugs combined. For instance, a patient treated with lithium and quetiapine is characterized by a PI of 1.265 [1.39 (PI for lithium) +1.14 (PI for quetiapine)/2 (number of drugs) =1.265]. Unfortunately, PI values are not available for all drugs used in relapse prevention of BD. Most noteworthy, no PI was assigned to valproate and carbamazepine as Popovic *et al.* [14] could not conduct NNT analyses due to lack of respective randomized controlled trials (RCTs). In conformity with the validation study, these drugs were therefore not included in our analyses [15]. Aside from the total PI, we calculated separate PIs for antipsychotics (quetiapine, aripiprazole, risperidone, olanzapine, ziprasidone) and mood stabilizers (lithium, lamotrigine).

### Statistical analysis

For statistical analysis of the data, we used the Statistical Package for Social Sciences (SPSS Inc., Chicago, Illinois, USA) software for Windows (version 21). Demographic, clinical, and treatment variables of the three groups (DPP, MPP and UPP) were compared using Chi Square Tests (categorical variables) or ANOVAs (continuous variables). All statistics were two-tailed. Bonferroni correction for multiple testing was applied. Since the Kolmogorov-Smirnov Test indicated no normal distribution of the data, Mann-Whitney-*U*-Test was used to analyze differences in mean PI between the two groups DPP and MPP. In order to ensure comparability with the original study [15], we omitted the UPP group from this analysis.

## Results

### Differences in clinical characteristics of BP with predominant polarity

Our sample consisted of 336 BP with diagnosis type BP I or II. 215 (63.9%) fulfilled the criteria of one predominant polarity (MPP or DPP). The remaining 121 (36.1%) BP with undetermined predominant polarity (UPP) were also included in the analysis. Within the predominant polarity sample, 169 (78.6%) patients were classified as DPP and 46 (21.4%) as MPP. Demographic and clinical characteristics of the sample are shown in Table 1.

**Table 1:** **Demographic and clinical characteristics of the bipolar sample according to predominant polarity**

	**DPP (N = 169)**	**MPP (N = 46)**	**Chi Square**	***p***_***adj***_^***2***^
	**N (50.3%)**	**N (13.7%)**		
Sex			5.49	.019
Female	116 (68.6)	23 (50)		
Male	53 (31.4)	23 (50)		
Subtype			31.45	<.000*
Bipolar I	80 (47.3)	43 (93.5)		
Bipolar II	89 (52.7)	3 (6.5)		
Family history of psychiatric disorders	138 (81.7)	35 (76.1)	0.71	.398
Family history of suicide	59 (34.9)	12 (26.1)	1.27	.259
Rapid cycling	19 (11.2)	3 (6.5)	0.88	.349
Psychotic symptoms	22 (13.0)	13 (28.3)	6.16	.013
First Episode Mania	11 (6.5)	30 (65.2)	80.75	<.000*
Mania with irritable mood	52 (30.8)	34 (73.9)	28.04	<.000*
Mixed Episodes	55 (34.6)	16 (39.0)	0.28	.597
Suicide attempts (life time)	68 (40.2)	15 (32.6)	0.89	.346
Substance abuse (life time)	18 (10.7)	10 (21.7)	3.93	.481
*Treatment*				
Lithium	74 (43.8)	30 (65.2)	6.65	.010
Other Mood Stabilizers^1^	63 (37.3)	26 (56.5)	5.52	.019
Antipsychotics (incl. quetiapine)	117 (69.2)	43 (93.5)	11.17	.001*
Antidepressants	121 (71.6)	14 (30.4)	26.22	<.000*
	Mean (SD)	Mean (SD)	*t*	*p*_*adj.*_^*2*^
Age	50.3 (14.8)	52.1 (12.2)	-.765	.451
Age of Onset	29.8 (11.8)	28.5 (10.4)	.667	.505
Age of first depression	29.7 (12.1)	31.6 (12.1)	-0.85	.396
Age of first mania	37.1 (14.5)	28.8 (11.0)	3.43	.001*
Duration of illness	20.5 (12.5)	23.6 (12.6)	-1.45	.148
Total number of episodes	11.1 (10.2)	10.7 (6.3)	0.282	.778
Number of manic episodes	2.3 (2.5)	8.3 (5.5)	-10.56	<.000*
Number of depressive episodes	8.6 (8.0)	2.4 (2.0)	5.26	<.000*
Number of mixed episodes	1.0 (2.8)	0.71 (1.2)	.736	.463

*p< .05.

DPP = depressive predominant polarity, MPP = manic predominant polarity, UPP = uncertain predominant polarity.

^1^(Valproate, Lamotrigine, Carbamazepine, Oxcarbazepine).

^2^We used bonferroni correction for multiple testing (23 hypotheses) for adjusting the p-values to 0.0022 (0.05/23).

We compared the demographic and clinical parameters between the two groups DPP and MPP in order to detect differences between BP with regard to their predominant polarity. After Bonferroni correction for multiple testing, we found that patients with MPP had significantly more often a diagnosis of BD-Type I (p < .000), while in the DPP group both subtypes were distributed equally. Irritability during mania was significantly more prevalent in patients with MPP (*p* < .000). Patients with MPP more often had a first episode of mania at illness onset (*p* < .000), and patients classified as MPP were younger when experiencing their first (hypo-) manic episode (*p* = .001), compared to patients with DPP. Psychotic symptoms were more prevalent in MPP (*p* = .013), and females were more often classified as DPP, while MPP was equally distributed in women and men (*p* = .019). However, due to Bonferroni correction both group differences did not reach significance.

### Polarity index and pharmacological treatment

Mann-Whitney-*U*-Tests were used to compare the PI of DPP and MPP (Table 2). No significant difference in total PI between DPP and MPP was observed. However, the PI of mood stabilizers was lower in patients with DPP compared to patients with MPP (*p* = .024), indicating a stronger antidepressant regimen in the DPP group. There was no difference in the PI of antipsychotics between groups. As there were no PI values for valproat and carbamazepine available, we conducted a subset analyses by excluding those patients treated with one of these mood stabilizers. However, again we found no significant difference in the total PI (*p* = .810), PI of mood stabilizers (*p* = .658) and PI of antipsychotics (*p* = .238) between DPP and MPP.

**Table 2:** **Mean Values and tests of differences of Polarity Indices in predominant polarity groups**

	**DPP**	**MPP**	**Kolmogorov-Smirnov Z**	***p***	**Mann-Whitney U-Test**	***p***
	**(N = 169)**	**(N = 46)**				
	Mean (SD)	Mean (SD)				
Total Polarity Index	1.95 (0.20)	2.17 (0.37)	5.947	.000***	3579.5	.407
Polarity Index AP	1.87 (0.23)	2.20 (0.52)	6.091	.000***	388.5	.997
Polarity Index MS	0.63 (0.50)	0.89 (0.96)	5.733	.000***	3118.5	.024*

*p< .05.

***p< .001.

DPP = depressive predominant polarity, MPP = manic predominant polarity, UPP = uncertain predominant polarity, AP = Antipsychotics, MS = Mood Stabilizers.

The pharmacological treatment of BP is given in detail in Table 3. Approximately half of the total sample was treated with lithium. Between groups, patients with MPP were more often medicated with lithium compared to patients with DPP or UPP. Furthermore, our results show that more patients with MPP were medicated with valproate compared to patients with DPP or UPP. However, these differences did not reach statistical significance after correction for multiple testing. 15.5% of the total bipolar sample were treated with first generation antispychotics (FGA), while second generation antipsychotics (SGA) were prescribed in 67.3% of all BP. Here, quetiapine was most commonly used. More than half of the total bipolar sample (58.9%) was medicated with antidepressants as add-on therapy, of which tricyclics and selective noradrenalin reuptake inhibitors (SNRIs) were most commonly used. 71.6% of patients with DPP were treated with mood stabilizers and antidepressants, compared to 30.4% of patients with MPP. Overall, 170 patients (50.6%) received polytherapy, distribution of which did not differ between the three groups. Patients with MPP significantly more often received a combination of lithium and another mood stabilizer (*p* < .000), or a combination of lithium and second generation antipsychotics, compared to patients with DPP and UPP (*p* < .000).

**Table 3:** **Frequency of prescribed drugs in the bipolar sample**

	**N**_**total**_ **= 336**	**DPP**	**MPP**	**UPP**	**Chi Square**	***p***_***adj***_^***1***^
	**(100%)**	**N = 169**	**N = 46**	**N = 121**		
		**(50%)**	**(14%)**	**(36%)**		
Mood Stabilizers						
Lithium	165 (49.1)	74 (43.8)	30 (65.2)	61 (50.4)	6.77	.034
Carbamazepine	10 (3.0)	2 (1.2)	4 (8.7)	4 (3.3)	7.14	.028
Lamotrigine	32 (9.5)	22 (13.0)	3 (6.5)	7 (5.8)	4.84	.089
Valproate	100 (29.8)	38 (22.5)	19 (41.3)	43 (35.5)	9.14	.010
Topiramate	0 (0.0)	0 (0.0)	0 (0.0)	0 (0.0)	-	-
FGA	52 (15.5)	16 (9.5)	15 (32.6)	21 (17.4)	15.31	<.000*
SGA	226 (67.3)	109 (64.5)	33 (71.7)	84 (69.4)	1.26	.532
Quetiapine	138 (41.1)	70 (41.4)	21 (45.7)	47 (38.8)	.66	.721
Aripiprazole	38 (11.3)	22 (13.0)	2 (4.3)	14 (11.6)	2.72	.256
Clozapine	18 (5.4)	5 (3.0)	4 (8.7)	9 (7.4)	3.96	.138
Risperidone	40 (11.9)	13 (7.7)	7 (15.2)	20 (16.5)	5.81	.055
Olanzapine	21 (6.3)	14 (8.3)	2 (4.3)	5 (4.1)	2.40	.301
Ziprasidone	1 (0.3)	1 (0.6)	0 (0.0)	0 (0.0)	0.99	.609
Pregabalin	17 (5.1)	8 (4.7)	1 (2.2)	8 (6.6)	1.44	.486
Antidepressants	198 (58.9)	121 (71.6)	14 (30.4)	63 (52.1)	28.99	<.000*
TCAs	91 (27.1)	56 (33.1)	5 (10.9)	30 (24.8)	9.58	.008
IMAOs	3 (0.9)	3 (1.8)	0 (0.0)	0 (0.0)	2.99	.224
SSRIs	32 (9.5)	17 (10.1)	2 (4.3)	13 (10.7)	1.70	.428
SNRIs	75 (22.3)	49 (29.0)	3 (6.5)	23 (19.0)	11.73	.003
Other	11 (3.3)	6 (3.6)	1 (2.2)	4 (3.3)	0.217	.897
Polypharmacy (total)	170 (50.6)	94 (55.6)	23 (50)	53 (43.8)	3.94	.139
Lithium + other MS	46 (13.7%)	17 (10.1%)	16 (34.8%)	13 (10.8%)	19.98	<.000*
Lithium + SGAs	119 (35.5%)	48 (28.4%)	28 (60.9%)	43 (35.8%)	16.64	<.000*

*p< .05.

DPP = depressive predominant polarity, MPP = manic predominant polarity, UPP = undetermined predominant polarity.

MS = Mood Stabilizer, FGA = first generation antipsychotics, SGA = second generation antipsychotics, MAOs = Monoamine Oxidase Inhibitor.

^1^We used bonferroni correction for multiple testing (20 hypotheses) for adjusting the p-values to 0.0025 (0.05/20).

## Discussion

In the present study we investigated a sample of 336 BP with regard to their predominant polarity, which is defined as twice the number of previous episodes of one pole over the other. We aimed to identify clinical characteristics associated with predominant polarity. Furthermore, we attempted to validate the recently published Polarity Index [14], a metric algorithm which expresses the efficacy profile of a given drug in the maintenance treatment of BD.

Approximately half of our sample was classified as predominantly depressive (50.3%), 13.7% of patients as predominantly manic, and 36% of patients had no defined polarity. We found that the diagnosis type BP-I was more prevalent in patients with MPP compared to patients with DPP. These findings are in line with previous studies about predominant polarity and polarity of first episode [4], given that patients with a manic episode at illness onset more often receive the diagnosis type BP-I [2]. A recent follow-up study by Gonzalez-Pinto *et al.* [7] demonstrated that patients with DPP overall had more episodes, more hospitalizations, and more suicide attempts. However, we found no significant differences in number of previous episodes and suicidality between the three groups. At onset of illness, manic episodes were more frequent in patients classified with MPP compared to patients with DPP. This result, again, is in line with previous work demonstrating that polarity of first episode predicts the polarity of subsequent episodes over time [21,22]. In addition, patients with MPP were significantly younger when experiencing their first manic episode compared to patients classified as DPP. In line with previous studies, our results show that DPP was more prevalent in women, while MPP was equally distributed in women and men [5,23]. Regarding family history of psychiatric disorders, the literature showed an association with DPP [7], however we could not confirm this finding in our sample.

Regarding the Polarity Index, we could not replicate the results by Popovic *et al.* [15]. In our sample, the PI of maintenance treatment did not differ significantly between patients with DPP and MPP. Therefore, we could not validate the Polarity Index as a numeric expression of the efficacy of maintenance treatment under naturalistic conditions. Only when mood stabilizers (lithium and lamotrigine) were considered separately were we able to detect a difference: Patients with DPP had a lower PI of mood stabilizers compared to patients with MPP. Furthermore, patients with DPP were treated significantly more often with lamotrigine and antidepressants than patients with MPP. In turn, lithium, valproate, carbamazepine, risperidone and FGAs were more frequently prescribed in MPP compared to DPP. Hence, our bipolar sample received an evidence-based treatment for relapse prevention, supported by pertinent guidelines such as those published by the German Association for Psychiatry, Psychotherapy and Psychosomatics (DGPPN) [24] or the Canadian Network for Mood and Anxiety Treatments [25]. It is therefore possible that the difference in results may be due to different prescription patterns in the present sample than in the Spanish sample. In our sample, valproate, quetiapine, and aripiprazole were prescribed considerably more often than in the study by Popovic *et al.* [15]. In contrast, risperidone, olanzapine, and carbamazepine, which are often used in maintenance treatment by the Spanish colleagues, were rarely prescribed in our sample. Furthermore, antidepressants as add-on therapy were administered more often in our sample. Due to the naturalistic study design, our study sample also differed in sociodemographic and clinical parameters from the sample of Popovic *et al.* [15]. For instance, diagnosis type BP-II was overrepresented in our sample. Additionally, the Spanish sample consisted of 44.4% of patients classified as MPP, compared to only 21.4% in our sample. Therefore, despite using a similar study design, different sample characteristics and prescription patterns may be attributable for the divergent results in our German sample.

A further factor hindering validation might be that no PI was assigned to valproate due to missing RCTs. Indeed, 30% of our patients were medicated with valproate, compared to only 17% in the sample of Popovic *et al.* [15]. The exclusion of a compound used in almost a third of the patients therefore might have distorted our results. In a similar vein, antidepressants do not have a PI either, despite playing an undervalued role in the maintenance treatment of BD [26], and consequently were not considered in the present analysis. Moreover, psychosocial interventions were also not considered for analysis because information was not available about how many patients of our sample received psychoeducation or psychotherapy in the past. As Popovic *et al.* [17] demonstrated, psychosocial interventions differ in their depression- or mania-preventive effects and can be evaluated by the PI. We therefore conclude that the unavailability of a PI for several frequently used compounds in maintenance treatment in BD might account for our non-validation. Since we still consider the PI a clinically useful measure, an effort should be made to assign a PI to all substances used in the treatment of BD. In the perspective of evidence-based medicine, the PI could act in addition to clinical interviews to provide a high standard of patient care.

However, the PI has some general limitations which should be considered. First of all, the index does not reflect pharmacodynamic interactions in the case of polypharmacy, as it is only the numeric mean of the PIs of the individual drugs, regardless of their dosage or possible interactions. Furthermore, the PIs of drugs used in maintenance treatment are calculated according to current available RCT studies. Up to now, well-designed RCTs are rare, especially for mood stabilizers like valproate and carbamazepine [16]. Therefore, the PI is subject to continuous change with each new study. Other open questions concern the maintenance treatment of patients without predominant polarity (in the present sample, 36%), treatment of first-episode patients, and how mixed states should be classified. Furthermore, sub-syndromal depressive symptoms, which are prevalent during remission in BD, are not taken into account in the classification of predominant polarity [6]. Despite these methodological shortcomings, the idea of assigning a PI to a given drug and following rule-based treatment upon the predominant polarity seems reasonable and worthwhile; although we assume that psychiatrists follow these rules intuitively, a formal description bears advantages especially for education purposes.

The present study design also has methodological limitations. Our data consisted of retrospective self-reports on previous episodes from BP, their relatives, and clinical data. These are partially subjective, thus reducing the reliability of the data. Especially hypomanic episodes are often not remembered well or tend to be misinterpreted by patients. Furthermore, the subgroup of patients with MPP was relatively small, which could explain the absence of statistical differences in PI values between patients with DPP and MPP.

## Conclusions

To summarize, we found that predominant polarity was associated with several clinical variables like sex, diagnosis subtype, irritability during mania, polarity at illness onset and age of onset. However, we could not validate the Polarity Index suggested by Popovic *et al.* [15] in our bipolar sample. Despite evidence of different treatment strategies in patients with depressive or manic predominant polarity, we found no significant differences in PI values. Nevertheless, psychopharmacological strategies must be adapted to the most frequently observed polarity. The reason for the negative finding in this study might well be that for some frequently used drugs in maintenance treatment of BD no PI value is available. Furthermore, we had different sample characteristics and prescription patterns compared to Popovic et al. [15]. Therefore, despite the failed validation of the PI in our sample, we suggest the PI as a helpful orientation in decision-making of maintenance treatment.
